# Association between 1p11-rs11249433 Polymorphism and Breast Cancer Susceptibility: Evidence from 15 Case-Control Studies

**DOI:** 10.1371/journal.pone.0072526

**Published:** 2013-08-15

**Authors:** Sheng Wu, Jungang Cai, Hong Wang, Hongwei Zhang, Weige Yang

**Affiliations:** 1 Department of General Surgery, Zhongshan Hospital, Qingpu Branch, Fudan University, Shanghai, People’s Republic of China; 2 Department of General Surgery, Zhongshan Hospital, Fudan University, Shanghai, People’s Republic of China; University of North Carolina School of Medicine, United States of America

## Abstract

Genome-wide association studies have identified SNP rs11249433 at chromosome 1p11 as a new breast cancer (BC) susceptibility locus in populations of European descent. Since then, the relationship between 1p11- rs11249433 and breast cancer has been reported in various ethnic groups; however, these studies have yielded inconsistent results. To investigate this inconsistency, we performed a meta-analysis of 15 studies involving a total of 90,154 cases and 137,238 controls for 1p11-rs11249433 polymorphism to evaluate its effect on genetic susceptibility for breast cancer. An overall random effects odds ratio of 1.09 (95% CI: 1.06-1.12, P<10^-5^) was found for rs11249433-G variant. Significant results were also observed for heterozygous (OR=1.09, 95% CI: 1.05-1.12, P<10^-5^) and homozygote (OR=1.14, 95% CI: 1.08-1.21, P<10^-5^). There was strong evidence of heterogeneity, which largely disappeared after stratification by ethnicity. After stratiﬁed by ethnicity, significant associations were found among Caucasians. However, no significant associations were detected among East Asian and African populations. In addition, we found that rs11249433 polymorphism on 1p11 confer risk, exclusively for ER-positive tumors with per-allele OR of 1.13 (95% CI: 1.08-1.18; P <10^-5^) compared to ER-negative tumors of 1.01 (95% CI: 0.98-1.04; P=0.49). Similar results were also observed when stratified by PR status. Our findings demonstrated that rs11249433-G allele is a risk-conferring factor for the development of breast cancer, especially in Caucasians.

## Introduction

Breast cancer (BC) is one of the most common malignancies among women worldwide [1]. Epidemiologic investigation of breast cancer has identiﬁed a number of environmental and lifestyle risk factors [2]. Breast cancer is nearly twice as frequent in ﬁrst-degree relatives of women with the disease than in relatives of women without this history, suggesting an important contribution of inherited susceptibility [3]. Furthermore, twin studies indicate that approximately 27% of breast cancer risk is due to inherited susceptibility [4]. Despite much investigation, only a few risk genes have been identified. These include rare, high-penetrance germline mutations segregating in high-risk pedigrees, notably in the BRCA1 and BRCA2 genes [5,6] and a handful of rare susceptibility variants with lower penetrance identiﬁed in DNA repair and apoptosis genes [7–11]. However, these genes account for less than 5% of overall breast cancer patients and most of the risk is likely to be attributable to more low-penetrance genetic variants [12]. Genome-wide association studies (GWAS) have identified multiple new common genetic variants associated with breast cancer risk in the general population. Common genetic variant rs11249433 at chromosome 1p11, 2 has been identified as a new hotspot for breast cancer susceptibility by a recent GWA study [13]. Associations between the 1p11-rs11249433 polymorphism and breast cancer have been independently replicated by subsequent studies; however, a proportion of them have yielded apparently conflicting results. Published studies have generally been restricted in terms of sample size and ethnic diversity, and individual studies may have insufﬁcient power to reach a comprehensive and reliable conclusion. To help clarify the inconsistent ﬁndings, we conducted a comprehensive meta-analysis to quantify the overall risk of 1p11-rs11249433 polymorphism on developing breast cancer.

## Materials and Methods

### Literature search strategy and inclusion criteria

Eligible literatures published before the end of March 2013 were identified by a search of PUBMED, EMBASE, and ISI web of science and CNKI (China National Knowledge Infrastructure) databases using combinations of the following keywords: “breast cancer,’’ ‘‘malignant breast neoplasm,’’ ‘‘1p11,’’ ‘‘rs11249433,’’ ‘‘polymorphism,’’ “variation”, without restriction on language. All references cited by identiﬁed eligible studies and previous reviews were scrutinized to ﬁnd additional work not indexed by PubMed.

Articles were included in this meta-analysis if they (1) examined the hypothesis that 1p11-rs11249433 polymorphism was associated with breast cancer risk, (2) followed a case-control or cohort study design, (3) identified breast cancer cases histologically or pathologically, and (4) provided sufficient information on genotype/allele counts between cases and controls to estimate the odds ratio (OR) and the corresponding 95% confidence interval (95% CI).

### Data extraction

Information was carefully extracted from all eligible publications independently by two of the authors according to the inclusion criteria listed above. The following variables were extracted from each study: the ﬁrst author, published year, study design, geographic area, ethnicity, mean age of cases and controls, case-control match status, deﬁnition and numbers of cases and controls, source of controls, genotyping method, frequency of genotypes, and Hardy-Weinberg equilibrium (HWE) in controls. Relevant clinical characteristics included estrogen receptor (ER) status, progesterone receptor (PR) status, and tumor grade. Review reports from the two were then compared to identify any inconsistency, and differences were resolved by further discussion among all authors. Studies with different ethnic groups were considered as individual studies for our analyses.

### Quality assessment: extended-quality score

For association studies with inconsistent results on the same polymorphisms, the methodological quality should be assessed by appropriate criteria to limit the risk of introducing bias into meta-analyses or systematic reviews. A procedure known as ‘extended-quality score’ has been developed to assess the quality of association studies. The procedure scores each paper categorizing it as having ‘high’, ‘median’ or ‘poor’ quality. Detailed procedure of the quality assessment was previously described [14].

### Statistical methods

Deviation from HWE for controls was examined by χ^2^ tests with 1 degree of freedom. The strength of association between 1p11-rs11249433 polymorphism and breast cancer risk was assessed by OR with the corresponding 95% CI. The per-allele OR of the risk allele was compared between cases and controls. Then, we estimated the risks of the heterozygous and homozygous genotypes on BC compared with the wild-type homozygote. Cochran’s Q statistical test and I^2^ were performed to assess possible heterogeneity between the individual studies, and thus to insure that each group of studies was suitable for meta-analysis [15]. Random-effects and ﬁxed-effect summary measures were calculated as inverse-variance–weighted average of the log odds ratio [16]. 95% CIs were constructed using Woolf’s method [17]. The results of random-effects summary were reported in the text because it takes into account the variation between studies. Sources of heterogeneity were investigated by stratified meta-analyses based on ethnicity, sample size (No. cases ≥1000 or, <1000), ER (ER+ vs. ER-) and PR (PR+ vs. PR-) status. Ethnic group was defined as East Asians (i.e., Chinese, Japanese, and Korean), Caucasians (i.e. people of European origin), and Africans (i.e., people of African origin). Publication bias was assessed with the funnel plot [18] and Egger test [19]. Sensitivity analysis was performed by removing each individual study in turn from the total and re-analyzing the remainder. This procedure was used to ensure that no individual study was entirely responsible for the combined results. The analyses were carried out by using the STATA software version 10.0 (Stata Corporation, College Station, TX). The type I error rate was set at 0.05. All P-values were two-tailed.

## Results

### Characteristics of included studies

Study selection process was shown in Figure S1. A total of 15 studies with 90,154 cancer cases and 137,238 controls were retrieved based on the search criteria for BC susceptibility related to the 1p11-rs11249433 polymorphism [13,20–33]. In addition, all studies indicated that the frequency distributions of genotypes in the controls were consistent with Hardy–Weinberg equilibrium. The extended-quality scores ranged from 5 to 8, and 3 studies were given median quality, whereas 12 were given high quality. No ‘poor quality’ study was found. The main study characteristics were summarized in Table 1.

**Table 1:** Characteristics of studies included in a meta-analysis of the association between 1p11-rs11249433 and BC.

Study	Year	Ethnicity	No. of cases/controls	RAF in cases/controls	Genotyping method	Source of controls	Quality score
Thomas [13]	2009	American, Polish	6294/7247	0.42/0.38	SNP Array, TaqMan	GP	High
Bhatti [20]	2010	American	774/989	0.41/0.38	TaqMan	GP	Median
Long [21]	2010	Chinese	2044/2054	0.03/0.03	SNP Array, iPLEX	GP	High
Figueroa [22]	2011	European, Australian, American, Canadian, Chinese	46036/46930	0.27/0.24	TaqMan, iPLEX	GP, HP	High
Chen [23]	2011	American	3016/2745	0.13/0.13	SNP Array	GP	High
Antoniou [24]	2011	European, Australian, American, Canadian	9006/8155	0.41/0.40	TaqMan, iPLEX	GP	High
Hutter [25]	2011	American	316/7484	0.17/0.16	SNP Array	GP	High
Jiang [26]	2011	Chinese	1766/1853	0.04/0.03	TaqMan	GP	Median
Campa [27]	2011	American, European	8360/11513	0.43/0.40	TaqMan	GP	High
Stevens [28]	2011	European, Australian, American	2976/4968	0.40/0.41	iPLEX	GP	High
Li [29]	2011	Swedish, Finn	1557/4584	0.40/0.38	SNP Array	GP	High
Sueta [30]	2012	Japanese	697/1394	0.03/0.02	TaqMan	HP	Median
He [31]	2012	European, American	3683/34174	0.46/0.44	TaqMan	GP	High
Huo [32]	2012	Nigerian	1509/1383	0.10/0.10	GoldenGate	GP	High
Kim [33]	2012	Korean	2257/2052	0.04/0.04	SNP Array, TaqMan	GP	High

RAF: risk allele frequency, GP: general population, HP: hospital patient

### Association between 1p11-11249433 and breast cancer

**Figure 1:**
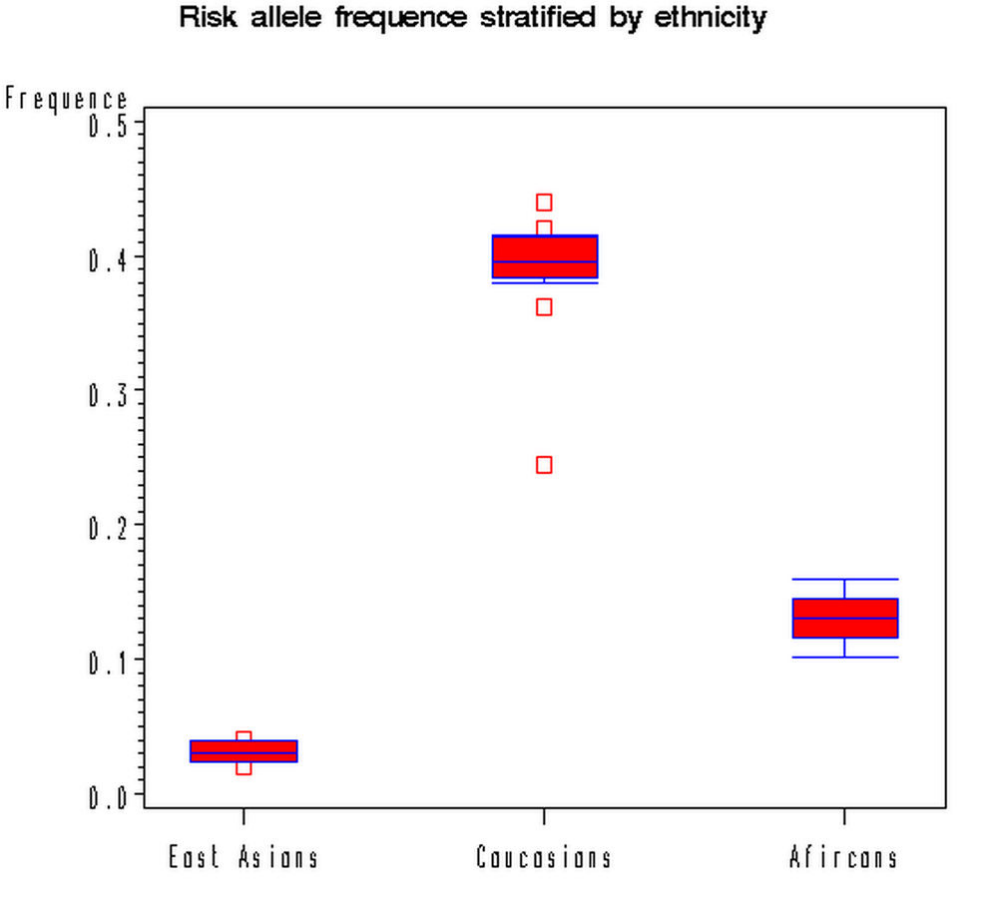
Frequencies of the risk allele of 1p11-rs11249433 polymorphism among controls stratiﬁed by ethnicity.

There was a wide variation in the G allele frequency of the rs11249433 polymorphism among the controls across different ethnicities, ranging from 0.02 to 0.44 (Table 1). For Caucasian controls, the G allele frequency was 0.40 (95% CI: 0.37-0.45), which was higher than that in East Asian controls (0.03; 95% CI: 0.01-0.06), and African controls (0.12; 95% CI: 0.10-0.16) (Figure 1).

**Figure 2:**
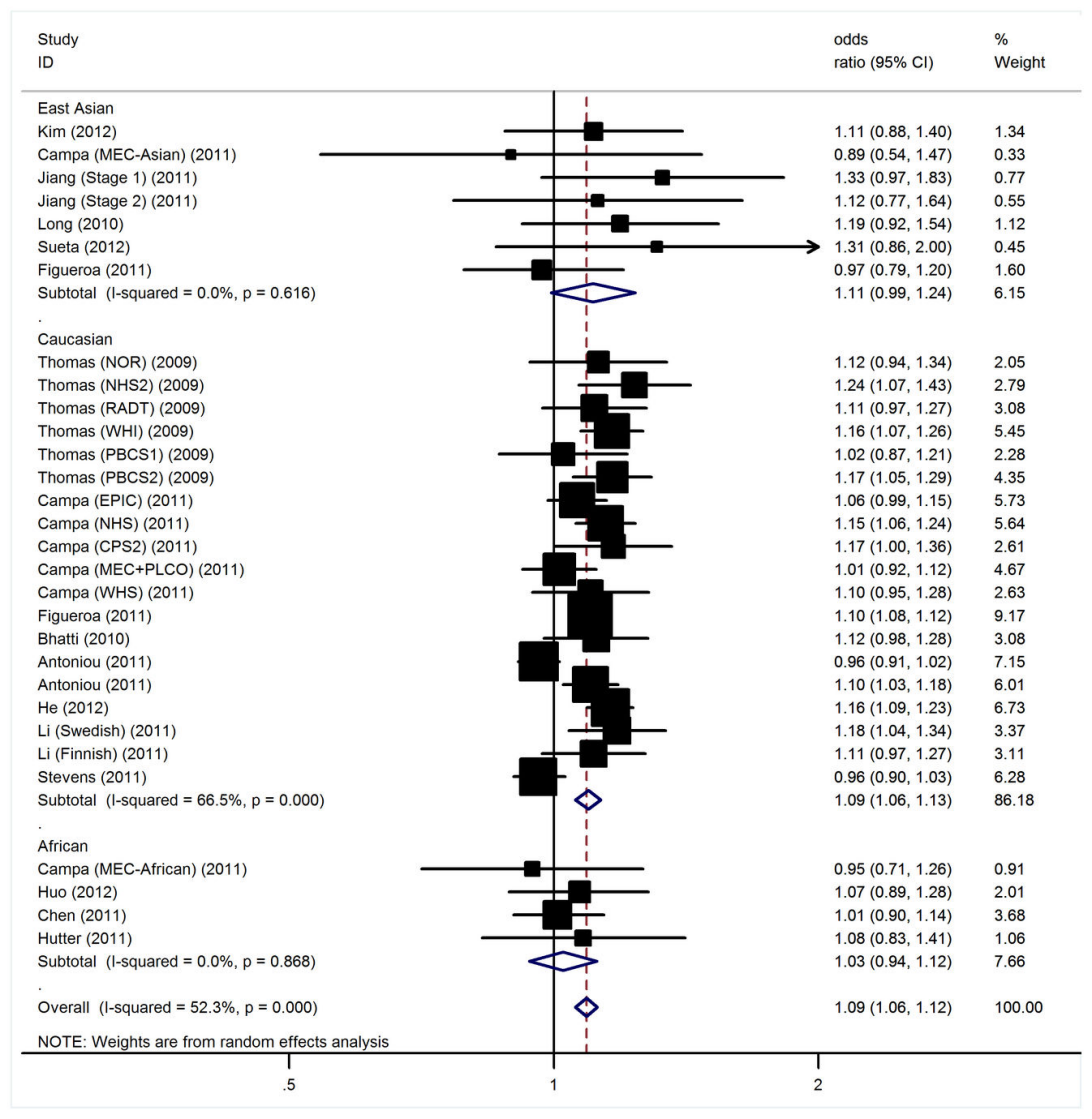
Forest plot for association of 1p11-rs11249433 polymorphism and BC risk.

For BC risk and the rs11249433 polymorphism, our meta-analysis gave an overall OR of 1.09 (95% CI: 1.06-1.12, P < 10^-5^; Figure 2) with statistically signiﬁcant between-study heterogeneity. Signiﬁcantly increased BC risks were also found for those heterozygous (OR = 1.09, 95% CI: 1.05-1.12; P < 10^-5^) and homozygous for the mutant allele (OR = 1.14, 95% CI: 1.08-1.21; P < 10^-5^) when compared with the wild type genotype.

In view of significant heterogeneity and to seek for its potential sources, we performed a panel of subgroup analyses on ethnicity and sample size. When studies were stratiﬁed for ethnicity, signiﬁcant risks were found among Caucasians in all comparisons (G allele: OR = 1.09, 95% CI: 1.06-1.13, P<10^-5^; heterozygous: OR = 1.10, 95% CI: 1.05-1.14, P<10^-5^; homozygote: OR = 1.16, 95% CI: 1.09-1.23, P<10^-5^). However, we failed to detect any association to BC susceptibility for East Asians and Africans in all genetic models (Table 2). In considering sample size subgroups, the OR was 1.13 (95% CI: 1.08-1.18, P <10^-4^) in small studies compared to 1.07 (95% CI: 1.03-1.12, P <10^-4^) in larger studies.

**Table 2:** Results of meta-analysis for 1p11-rs11249433 polymorphism and BC risk.

Overall and subgroups analyses	No. of cases/controls	G allele				Heterozygous				Homozygote			
		OR (95%CI)	P (Z)	P (Q)	I^2^	OR (95%CI)	P (Z)	P (Q)	I^2^	OR (95%CI)	P (Z)	P (Q)	I^2^
Overall	90154/137238	1.09 (1.06-1.12)	<10^-5^	<10^-5^	52%	1.09 (1.05-1.12)	<10^-5^	0.04	33%	1.14 (1.08-1.21)	<10^-5^	<10^-5^	66%
Ethnicity													
East Asian	10767/10366	1.11 (0.99-1.24)	0.07	0.63	0%	1.09 (0.98-1.22)	0.12	0.67	0%	1.12 (0.95-1.31)	0.17	0.28	19%
Caucasian	74145/114828	1.09 (1.06-1.13)	<10^-5^	<10^-5^	67%	1.10 (1.05-1.14)	<10^-5^	0.004	53%	1.16 (1.09-1.23)	<10^-5^	<10^-5^	74%
African	5242/12044	1.03 (0.94-1.12)	0.58	0.37	0%	1.03 (0.94-1.13)	0.55	0.95	0%	1.03 (0.89-1.19)	0.71	0.32	14%
Sample size													
<1000	9825/21877	1.13 (1.08-1.18)	<10^-5^	0.91	0%	1.13 (1.07-1.20)	<10^-5^	0.88	0%	1.18 (1.10-1.27)	<10^-4^	0.40	5%
≥1000	80329/115361	1.07 (1.03-1.12)	<10^-4^	<10^-5^	72%	1.08 (1.03-1.12)	0.001	0.004	56%	1.12 (1.04-1.20)	<10^-5^	<10^-5^	79%

**Figure 3:**
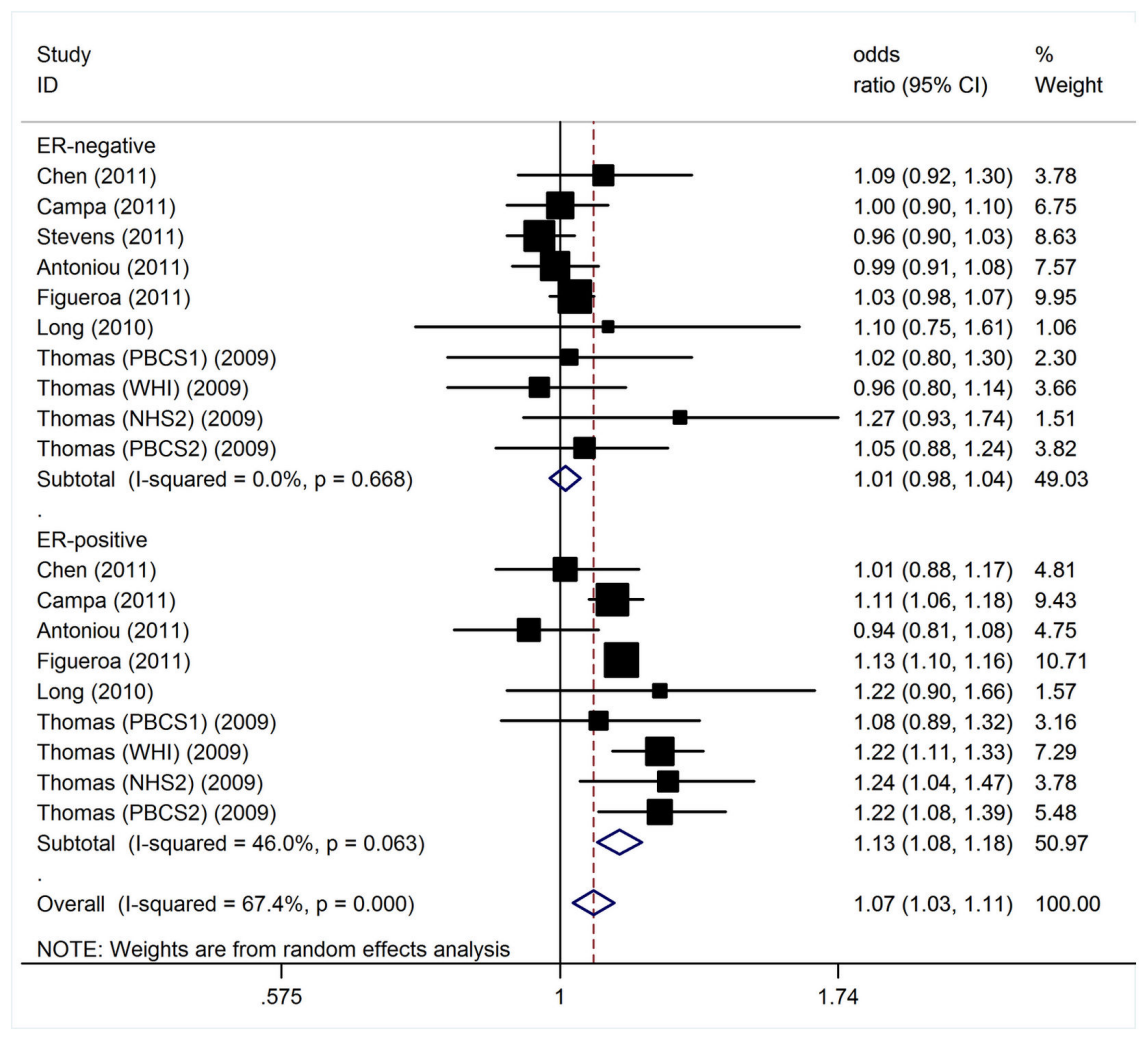
Per-allele odds ratios and 95% conﬁdence intervals for the association between 1p11-rs11249433 and BC risk by ER status.

The data on alleles of the polymorphism among cases stratiﬁed by ER status were available in 7 studies (including 37,514 cancer cases and 66,665 controls). We found that SNP rs11249433 on 1p11 confers risk preferentially for ER+ tumors [OR=1.13, 95% CI: 1.08-1.18, P (Z) < 10^-5^, P (Q) = 0.06]. However, no significant association was detected for ER- tumors [OR=1.01, 95% CI: 0.98-1.04, P (Z) = 0.49, P (Q) = 0.67] (Figure 3). Similar results were also found for PR+ breast cancer [per-allele OR = 1.13, 95% CI: 1.10-1.16, P (Z) < 10^-5^, P (Q) = 0.99], compared to PR- tumors with per-allele OR of 1.04 [95% CI: 0.97-1.12, P (Z) = 0.30, P (Q) = 0.01; Figure S2].

### Sensitivity analyses and publication bias

Sensitivity analysis was performed by excluding one study at a time. The results confirmed the significant association between the rs11249433 polymorphism and the risk of BC, with ORs and 95% CIs ranging from 1.08 (95% CI: 1.05-1.12) to 1.10 (95% CI: 1.07-1.13). Funnel plot and Egger’s test were performed to evaluate the publication bias of the literature reviewed. The shape of the funnel plots seemed symmetrical, suggesting no publication bias among the studies included (Figure S3). The Egger test provided further evidence that there was no publication bias among the studies included (P = 0.97).

## Discussion

The pathogenesis of the development and progression of breast cancer is far from being clear at present. Accumulated evidence suggests that it is a complex polygenic disorder for which genetic factors play an important role in disease etiology [4]. This is the most comprehensive meta-analysis examining the rs11249433 polymorphism on 1p11 and its relationship to susceptibility for BC. Its strength was based on the accumulation of published data giving greater information to detect signiﬁcant differences. In total, the meta-analysis involved 15 studies for BC which provided 90,154 cases and 137,238 controls. Our results suggested that the G allele of the 1p11-rs11249433 polymorphism is a risk factor for developing BC.

In the subgroup analysis, ethnicity was responsible for heterogeneity, and the ORs between different genetic models and sample size were consistent. The rs11249433 showed a positive association with BC in Caucasians; whereas no associations were found in East Asians and populations of African descent. There are several possible reasons for such ethnic differences. Firstly, the G allele frequency among controls was 0.03 in East Asians, 0.12 in Africans and 0.40 in Caucasian population, suggesting a possible role of ethnic differences in genetic backgrounds. Therefore, failing to identify any significant association in East Asians and Africans could be due to substantially lower statistical power caused by the relatively lower prevalence of G allele of 1p11-rs11249433. Secondly, study design or small sample size or some environmental factors may affect the results. ER status may be particularly important given that some GWAS findings are specific to ER-positive and ER-negative cancers [34,35] and because a higher proportion of African Americans are diagnosed with ER-negative cancers [36,37], resulting in risk differences. Furthermore, it is possible that variation at this locus has modest effects on BC, but environmental factors may predominate in its progress, and mask the effects of this variation. Specific environmental factors like lifestyle and hormone replacement therapy that have been already well studied in recent decades [1,4]. The unconsidered factors mixed together may cover the role of the polymorphism in East Asians and Africans. Moreover, a polymorphism may be in close linkage with another nearby causal variant in one ethnic population but not in another. 1p11-rs11249433 polymorphism may be in close linkage with different nearby causal variants in different populations.

Meta-analysis is often dominated by a few large studies, which markedly reduces the evidence from smaller studies. By considering sample size, significantly increased BC susceptibility in 1p11 rs11249433 variation was also found both in large and small studies for all genetic models. However, our results suggest an overestimation of the true genetic association by small studies.

ER status is known to affect prognosis of BC. Stratiﬁcation of tumors by ER status indicated that the 1p11 rs11249433 confer risk, preferentially for estrogen ER-positive tumors, with no risk for ER-negative BC. Results from subgroup analyses on ER status of tumors were in agreement with previous reports [35,38]. This tendency to be more strongly associated with the risk of ER-positive breast cancer has been observed for other clearly established susceptibility SNPs, notably FGFR2-rs2981582, 8q-rs13281615, and 5p-rs10941679 [35,36,39], perhaps reflecting the fact that they were initially identiﬁed by GWASs for which most of the case patients in the hypothesis-generating phases had ER-positive disease. However, the present ﬁndings support the notion that ER-positive and ER-negative tumors have different genetic components to their risks. Besides, we also found that the association appeared to be much stronger for PR-positive than the PR-negative breast cancer. Because ER and PR statuses are the major markers of breast cancer subtypes, these observations suggest that inherited risk variants of these subtypes may vary. The magnitude of the observed differences is small, and by themselves these ﬁndings are unlikely to have any immediate clinical implications. However, the observed differences provide clues to the biologic mechanisms that underpin tumor heterogeneity, which may ultimately lead to improved treatment and prevention.

The mechanism underlying the association of the 1p11 rs11249433 polymorphism with BC risk remains unknown. Recently, a study conducted by Fu et al. demonstrated that the expression of NOTCH2 differs in subgroups of breast tumors and by genotypes of the breast cancer-associated SNP rs11249433 [40]. The NOTCH pathway has key functions in stem cell differentiation of ER + luminal cells in the breast. Therefore, increased expression of NOTCH2 in carriers of rs11249433 may promote development of ER + luminal tumors. However, further studies are needed to investigate possible mechanisms of regulation of NOTCH2 expression by rs11249433 and the role of NOTCH2 splicing forms in breast cancer development.

Several potential limitations of the present meta-analysis should be taken into consideration. Firstly, our results were based on unadjusted estimates, while a more precise analysis should be conducted if all individual-level raw data were available, which would allow for the adjustment by other co-variants including age, cigarette consumption, alcohol drinking, menopausal status, and other lifestyle. Secondly, the subgroup meta-analyses on East Asian and African populations are based on a small number of studies with such information available. Nevertheless, the total number of subjects included in this part of the analysis comprises the largest sample size so far. As studies among the Non-Caucasians are currently limited, further studies including a wider spectrum of subjects should be carried to investigate the role of these variants in different populations. Thirdly, the single locus–based nature of meta-analysis precluded the possibility of gene-gene and gene-environment interactions, as well as haplotype-based effects, suggesting that additional studies assessing these aspects are necessary.

To the best of our knowledge, this study was the ﬁrst comprehensive meta-analysis to assess the relationship between the 1p11-rs11249433 polymorphism and BC susceptibility. Our meta-analysis showed that rs11249433 polymorphisms at 1p11 might be risk-conferring factor for the development of BC in Caucasians, but not in East Asians and Africans. As studies among these populations are currently limited, further studies including a wider spectrum of subjects to investigate the role of this variant in these populations will be needed.

## Supporting Information

Figure S1 **The flow chart of the included studies.**
(TIF)

Figure S2 **Per-allele odds ratios and 95% conﬁdence intervals for the association between 1p11-rs11249433 and BC risk by PR status.**
(TIF)

Figure S3 **Funnel plot of 1p11-rs11249433 polymorphism and BC risk.**
(TIF)

Checklist S1 (DOC)
