# Supplementary figures and images for: Association between 1p11-rs11249433 Polymorphism and Breast Cancer Susceptibility: Evidence from 15 Case-Control Studies
Source: PLoS One. 2013 Aug 15;8(8):e72526. doi: 10.1371/journal.pone.0072526 (PMC3744559; doi:10.1371/journal.pone.0072526)

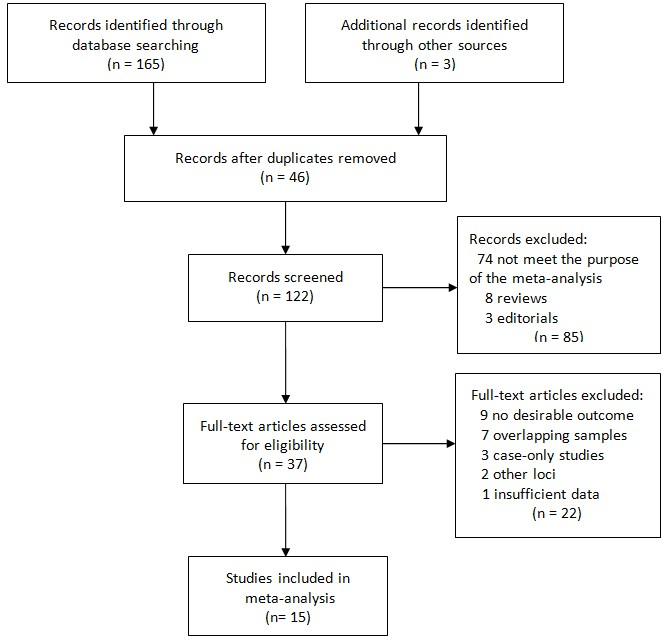

Supplement: Figure S1 — The flow chart of the included studies. (TIF) [file pone.0072526.s001.tif]

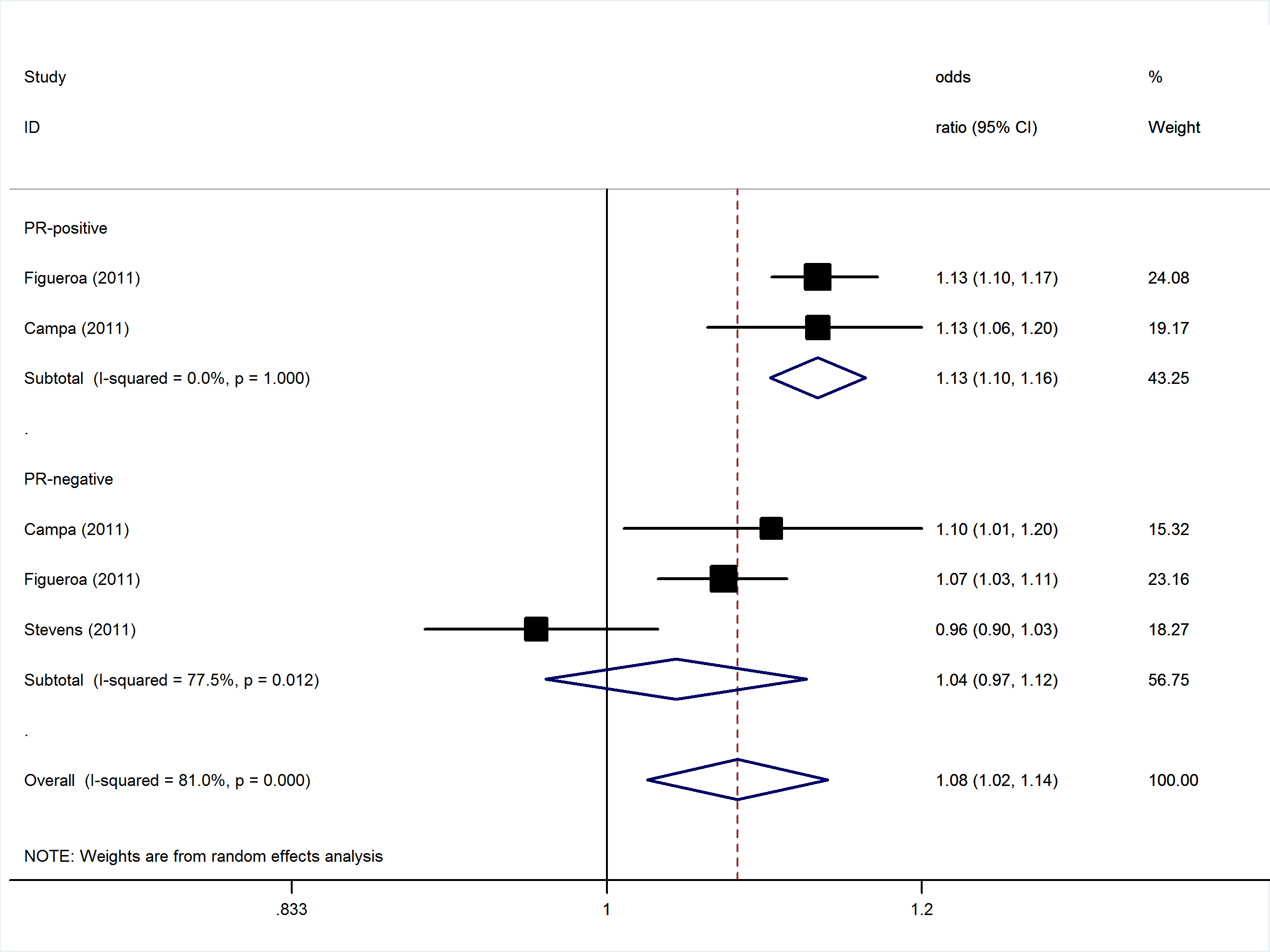

Supplement: Figure S2 — Per-allele odds ratios and 95% conﬁdence intervals for the association between 1p11-rs11249433 and BC risk by PR status. (TIF) [file pone.0072526.s002.tif]

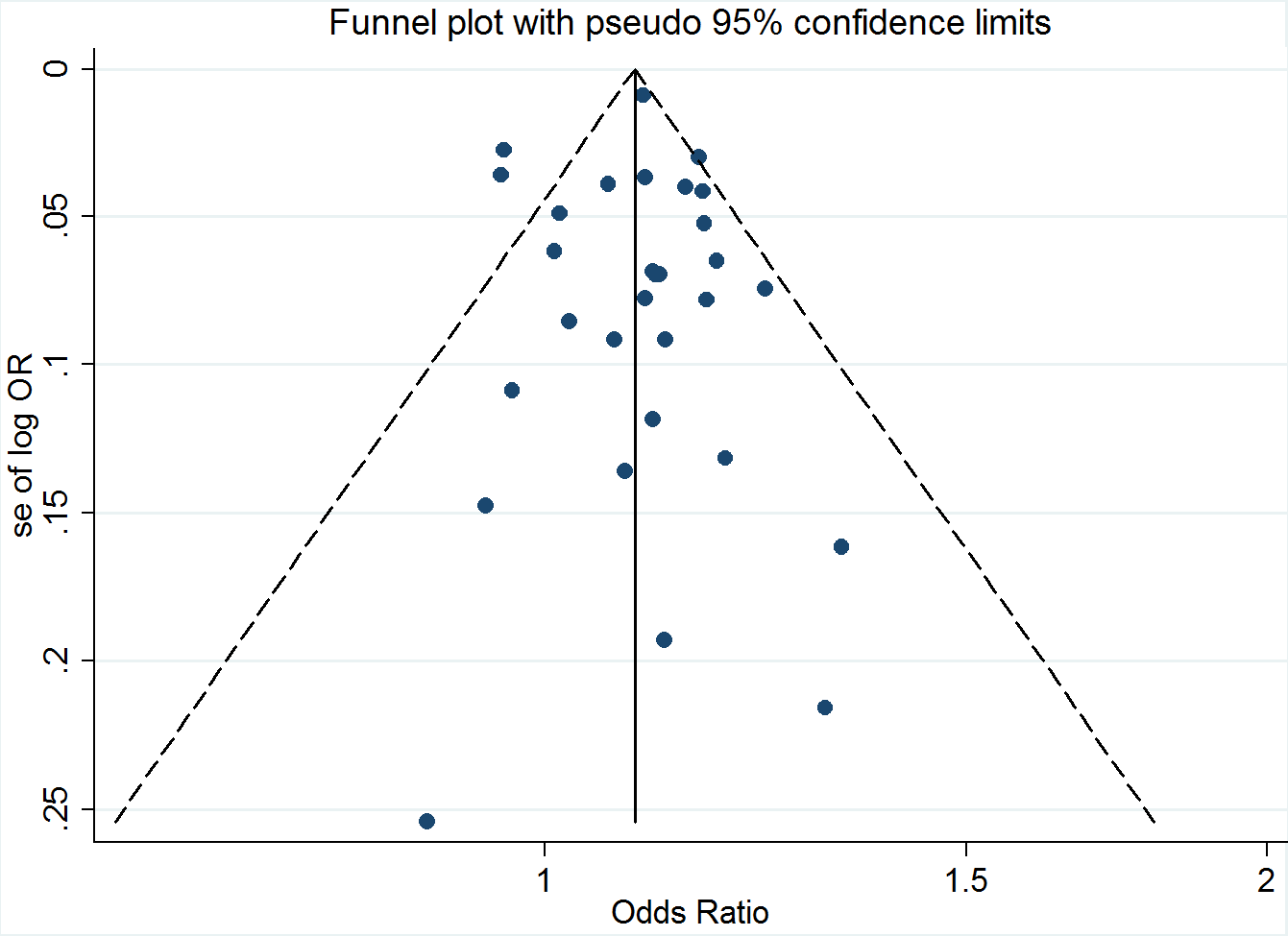

Supplement: Figure S3 — Funnel plot of 1p11-rs11249433 polymorphism and BC risk. (TIF) [file pone.0072526.s003.tif]
